# Case Report: Diagnostic dilemma: rebound thymic hyperplasia mimicking mediastinal relapse following chemoimmunotherapy in pediatric jejunal Burkitt lymphoma

**DOI:** 10.3389/fimmu.2026.1910294

**Published:** 2026-10-15

**Authors:** Meifang Lin, Zengwang Zhang, Hao Zhang, Dongdong Zhang

**Affiliations:** 1 Department of Oncology, Postgraduate Union Training Base of Xiangyang No. 1 People’s Hospital, Hubei University of Medicine, Xiangyang, China; 2 Department of Thoracic Surgery, Xiangyang Central Hospital, Hubei University of Arts and Science, Xiangyang, Hubei, China; 3 Department of Oncology, Xiangyang Central Hospital, Hubei University of Arts and Science, Xiangyang, Hubei, China

**Keywords:** Burkitt lymphoma, differential diagnosis, mediastinal mass, pediatric oncology, rebound thymic hyperplasia

## Abstract

**Background:**

Pediatric sporadic Burkitt lymphoma (BL) typically presents with gastrointestinal involvement; primary mediastinal disease is exceedingly rare. Rebound thymic hyperplasia (RTH) is a benign regenerative process following chemotherapy that can radiologically mimic mediastinal relapse, creating significant diagnostic challenges in post-treatment surveillance.

**Case presentation:**

An 8-year-old male was diagnosed with St. Jude stage III Burkitt lymphoma (BL) following the emergency resection of a jejunal mass. After receiving risk-adapted chemoimmunotherapy, he achieved a complete metabolic response. Three months post-treatment, routine surveillance computed tomography (CT) revealed a new anterior mediastinal soft tissue mass, magnetic resonance imaging (MRI) revealing T2 hyperintensity in the anterior mediastinal lesion without restricted diffusion, while subsequent PET/CT demonstrated mildly increased FDG uptake, raising immediate clinical concern for disease relapse. To rule out malignancy, a CT-guided percutaneous biopsy was performed. Histopathological analysis revealed normal thymic microarchitecture containing Hassall’s corpuscles. The final imaging and pathological findings supported a diagnosis of chemotherapy-induced rebound thymic hyperplasia (RTH).

**Conclusion:**

Cytotoxic therapy induces profound thymic involution, often followed by striking regenerative expansion upon treatment cessation. Recognizing RTH is paramount to avoid misdiagnosing neoplastic recurrence and administering unwarranted therapies. This case highlights need to integrate advanced imaging and tissue biopsy when evaluating new mediastinal opacities in pediatric lymphoma survivors.

## Introduction

Burkitt lymphoma (BL) is the most common mature B-cell non-Hodgkin lymphoma in pediatric oncology, typically presenting as an abdominal malignancy (1). Because primary or contiguous mediastinal involvement at initial diagnosis is exceptionally rare in BL (2), the emergence of a *de novo* anterior mediastinal mass during post-treatment surveillance strongly suggests disease relapse or a secondary malignancy. However, such masses frequently represent rebound thymic hyperplasia (RTH), a benign, physiological compensatory hypertrophy of the thymus following the cessation of cytotoxic chemotherapy (3, 4).

Although RTH is well-documented in pediatric Hodgkin lymphoma cohorts—occurring in 51.9% to 67.7% of survivors and typically emerging two to three months post-treatment (5), clinical awareness remains limited regarding mature B-cell lymphomas like BL. Because RTH clinically and radiologically mimics true disease recurrence, distinguishing this benign regenerative process from malignant progression poses a significant diagnostic challenge. Mischaracterization frequently exposes pediatric patients to unwarranted invasive procedures and potentially toxic salvage therapies.

Here, we present the case of a pediatric patient with primary jejunal BL who developed a *de novo* anterior mediastinal mass during routine surveillance, which was ultimately diagnosed as RTH. By detailing this diagnostic dilemma, we aim to heighten clinical awareness of RTH in BL, thereby improving post-treatment surveillance strategies and preventing unnecessary treatment-related morbidity.

## Case presentation

An 8-year-old boy presented to our hospital with a chief complaint of mid-upper abdominal pain. He had no significant past medical history. Physical and laboratory examinations upon admission were unremarkable. Computed tomography (CT) revealed a soft-tissue mass in the proximal jejunum located in the left upper quadrant, causing localized small bowel dilatation and fluid accumulation, accompanied by marked wall thickening and perilesional fat stranding (**Figure 1A**). The patient underwent emergent laparoscopy under general anesthesia, comprising a partial jejunectomy, end-to-end jejunojejunostomy, adhesiolysis, and small bowel plication. Histopathological examination of the resected specimen demonstrated a diffuse infiltration of medium-sized lymphoid cells extending through the entire bowel wall and into the vasculature. These cells exhibited sparse basophilic cytoplasm, round nuclei, multiple small nucleoli, and frequent mitotic figures. Under low-power magnification, abundant tingible-body macrophages were scattered among the neoplastic B cells, imparting a characteristic “starry-sky” pattern (**Figure 1B**). Immunohistochemistry (IHC) was positive for CD10, CD20, BCL-6, c-MYC (>90%), PAX-5, and P53, alongside a Ki-67 proliferation index approximating 100%. The tumor cells were negative for BCL-2, CD3, and terminal deoxynucleotidyl transferase (TdT). *In situ* hybridization for Epstein-Barr virus-encoded RNA (EBER) was also negative (**Figures 1C–L**). Fluorescence *in situ* hybridization (FISH) revealed a *MYC* gene rearrangement in the absence of *BCL2* or *BCL6* rearrangements. These findings confirmed the diagnosis of classic Burkitt lymphoma.

**Figure 1:**
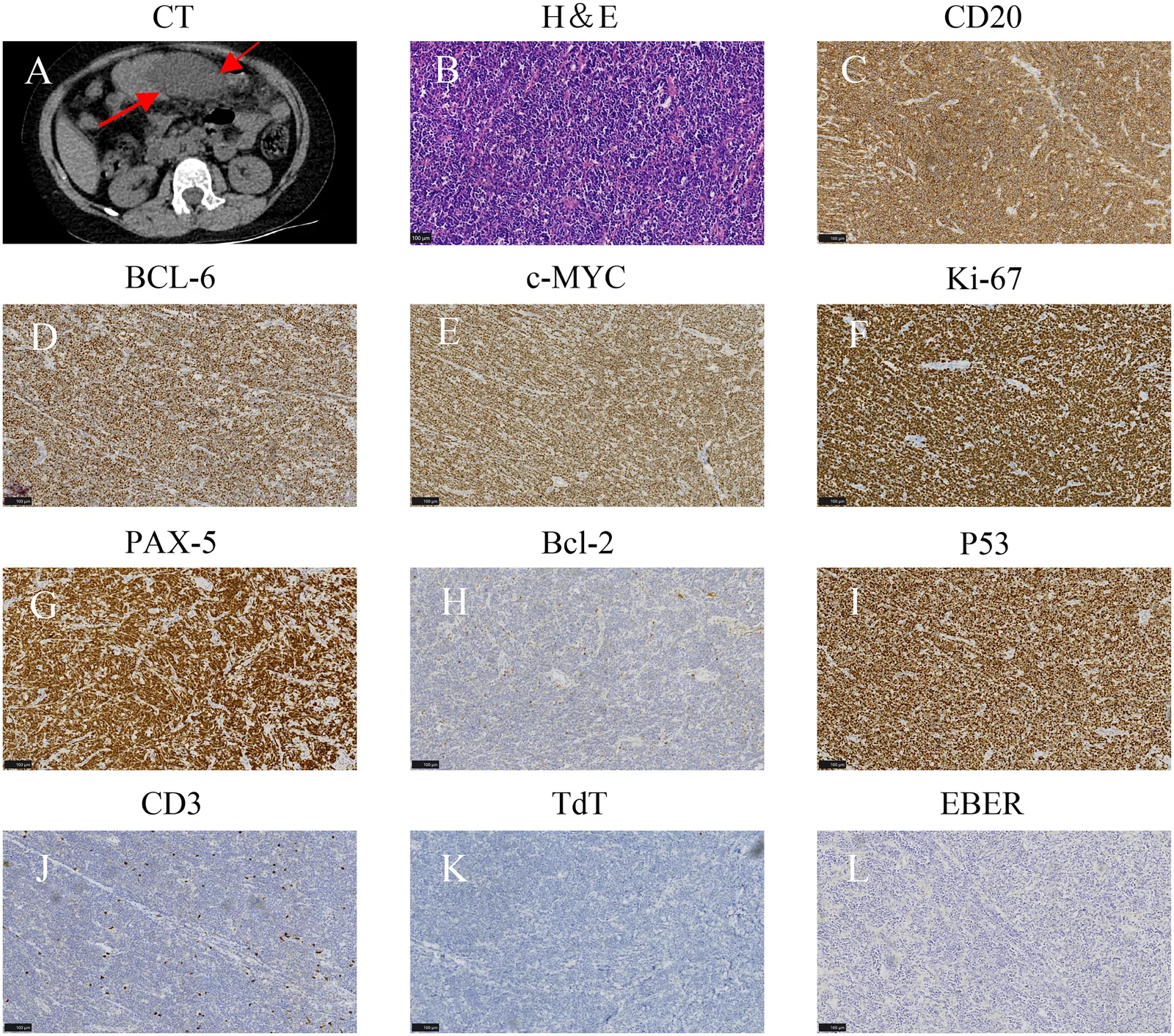
Initial presentation: Jejunal mass and immunophenotypic characterization confirming Burkitt lymphoma diagnosis. **(A)** Abdominal CT scan at diagnosis demonstrating a soft-tissue mass in the proximal jejunum (left upper quadrant) with localized small bowel dilatation, marked wall thickening, and perilesional inflammatory infiltration (fat stranding) (red arrows). **(B)** Hematoxylin and eosin (H&E) staining of the resected jejunal mass revealing a diffuse infiltration of medium-sized lymphoid cells characterized by basophilic cytoplasm, round nuclei, and multiple prominent small nucleoli. Tingible-body macrophages imparting a classic “starry-sky” pattern are scattered throughout the tissue (original magnification, ×200). **(C–G)** Immunohistochemistry demonstrating positive expression of B-cell and germinal center markers: **(C)** CD20 (diffuse membranous), **(D)** BCL-6 (nuclear), **(E)** c-MYC (strong, >90% of tumor cells), **(F)** Ki-67 (proliferation index ~100%), and **(G)** PAX-5 (nuclear). CD10 positivity was demonstrated but is not shown. These findings characterize the neoplasm as a mature B-cell lymphoma of germinal center origin with highly proliferative activity (original magnification, ×200). **(H)** BCL-2 staining showing complete negativity. **(I)** P53 exhibiting strong nuclear positivity in approximately 80% of tumor cells, indicating TP53 pathway involvement. **(J–L)** Negative staining for exclusion markers: **(J)** CD3 (T-cell lineage), **(K)** terminal deoxynucleotidyl transferase (TdT; excluding lymphoblastic lymphoma), and **(L)** EBER (EBV-encoded RNA *in situ* hybridization; classifying the case as EBV-negative sporadic Burkitt lymphoma).

Baseline whole-body positron emission tomography-computed tomography (PET-CT) was performed for staging. Imaging revealed extensive abdominopelvic peritoneal thickening alongside multiple hypermetabolic retroperitoneal and mesenteric lymph nodes. Hypermetabolic soft-tissue densities were also noted within the right rectus abdominis and abdominal wall subcutaneous fat. Increased metabolic activity was observed in bilaterally enlarged palatine tonsils, the nasopharyngeal roof, and bilateral cervical and supraclavicular lymph nodes (**Figures 2A–C**). Bone marrow aspiration and cerebrospinal fluid analyses were negative for lymphomatous involvement. The patient was classified as St. Jude stage III. According to the 2017 ChinaNet-Childhood Lymphoma (CNCL) protocol for mature B-cell non-Hodgkin lymphoma, the patient was stratified into the intermediate-risk group (Group B) (6). Postoperatively, the patient received risk-adapted sequential chemoimmunotherapy following the CNCL-2017 protocol (a modified LMB89-based regimen): a COP pre-phase (cyclophosphamide, vincristine, prednisone), followed by two COPADM courses at the COPADM3 dose level (cyclophosphamide, vincristine, prednisone, daunorubicin, and high-dose methotrexate 3 g/m²), two CYM courses (cytarabine and high-dose methotrexate), and three maintenance courses. Rituximab (375 mg/m² per dose, four doses total) was incorporated from the first COPADM course onward. Intrathecal prophylaxis—dual or triple therapy comprising dexamethasone and methotrexate, with or without cytarabine—was administered across the treatment courses for a total of ten injections (**Figure 3A**). At the completion of therapy, follow-up PET-CT confirmed a complete metabolic remission (CMR) (**Figures 2D–F**).

**Figure 2:**
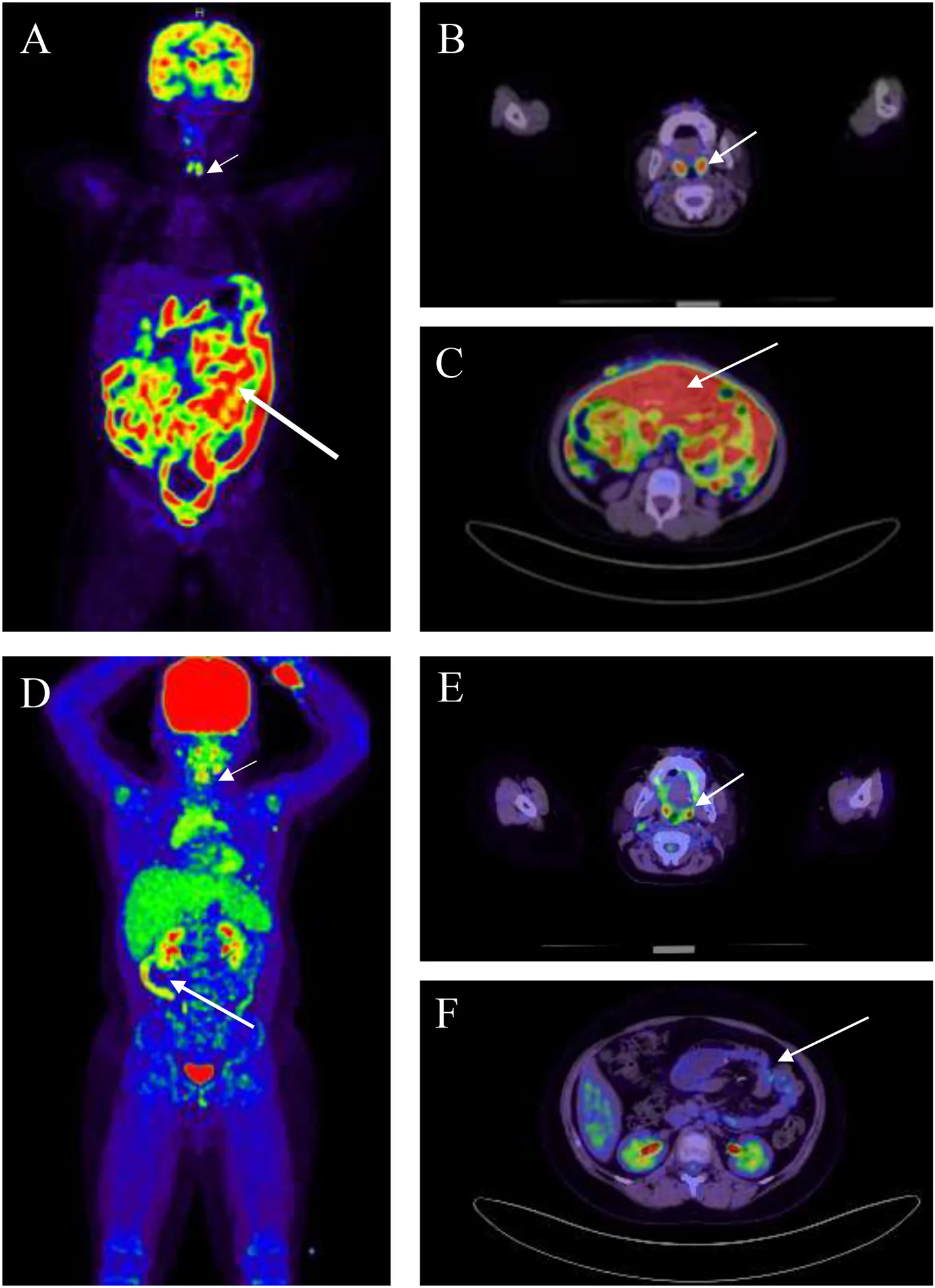
Comparison of pre-treatment and post-treatment PET-CT imaging demonstrating a complete metabolic response. Pre-treatment PET-CT (May 2025, baseline staging): **(A)** Whole-body maximum intensity projection (MIP) image revealing multifocal hypermetabolic disease: prominent FDG uptake in the neck (short arrow) and extensive, intense, heterogeneous FDG uptake throughout the abdomen and pelvis (long arrow), corresponding to peritoneal involvement and mesenteric lymphadenopathy. **(B)** Axial fused PET-CT image at the oropharyngeal level showing markedly increased FDG uptake in the bilateral palatine tonsils and cervical lymph nodes (arrow; SUVmax ~8.5), consistent with lymphomatous infiltration of Waldeyer’s ring. **(C)** Axial fused PET-CT image at the mid-abdominal level demonstrating intense, patchy FDG uptake within the mesentery and small bowel wall (arrow; SUVmax ~12.3), representing the jejunal primary tumor bed and peritoneal lymphomatous deposits. Additional hypermetabolic foci are present in the retroperitoneal and mesenteric lymph node chains. Post-treatment PET-CT (October 2025, end-of-treatment evaluation): **(D)** Whole-body MIP image demonstrating complete resolution of all previously identified hypermetabolic lesions. Only physiological FDG distribution is noted, including mild, symmetric uptake in the tonsillar region (short arrow) and renal/urinary excretion (long arrow). **(E)** Axial fused PET-CT at the corresponding cervical level showing near-complete normalization of FDG uptake in the previously involved tonsillar and cervical nodal regions (arrow). The residual mild, symmetric uptake (SUVmax 2.1) is indistinguishable from normal physiological background activity. **(F)** Axial fused PET-CT at the corresponding abdominal level demonstrating complete disappearance of the previously intense mesenteric and small bowel FDG uptake (arrow). No residual hypermetabolic foci are identified, confirming complete metabolic remission (CMR; Deauville score 1).

**Figure 3:**
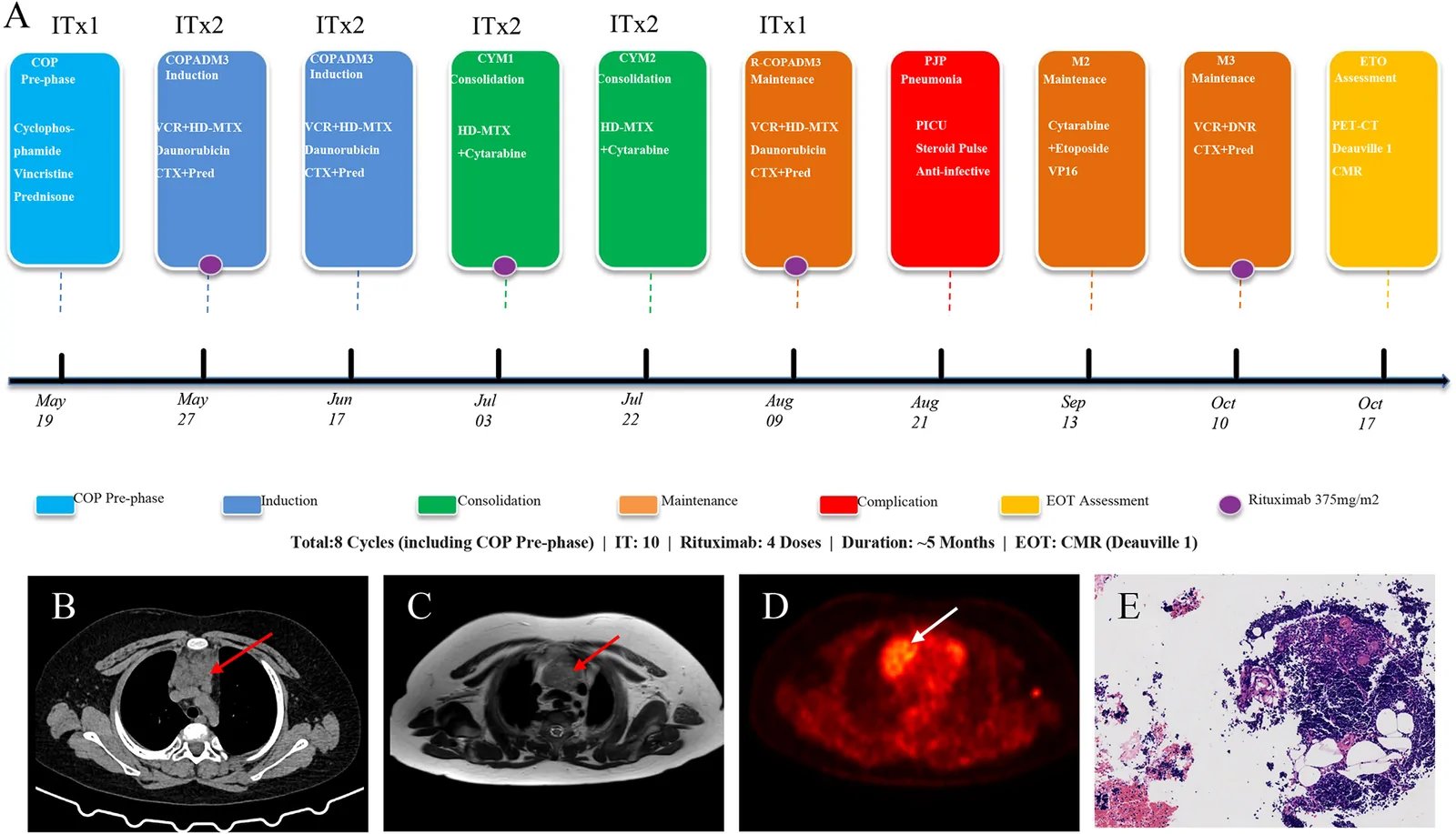
Treatment timeline and post-chemotherapy evaluation of rebound thymic hyperplasia (RTH). **(A)** Treatment timeline illustrating eight courses of the modified CNCL-2017 protocol based on LMB89 (Group B), comprising one COP pre-phase followed by seven additional chemotherapy courses (two COPADM3 induction courses, two CYM consolidation courses, and three maintenance courses) *Abbreviations:* CTX, cyclophosphamide; VCR, vincristine; Pred, prednisone; DNR, daunorubicin; HD-MTX, high-dose methotrexate (3 g/m²); Ara-C, cytarabine; VP16, etoposide; IT, intrathecal; CMR, complete metabolic remission; PJP, *Pneumocystis jirovecii* pneumonia. **(B)** Follow-up chest CT (January 2026) demonstrating a newly developed, well-circumscribed anterior mediastinal mass in the thymic region (arrow; 3.1 × 2.4 cm) with homogeneous attenuation. **(C)** Chest MRI revealing T2 hyperintensity in the anterior mediastinal lesion without restricted diffusion (arrow), strongly favoring benign thymic hyperplasia over malignant infiltration. **(D)** Follow-up PET-CT demonstrating mildly increased FDG uptake localized to the thymic region (SUVmax 3.3–3.5), from which the possibility of lymphoma relapse could not be excluded. **(E)** CT-guided core needle biopsy specimen (H&E, ×200) showing thymic tissue fragments with preserved Hassall corpuscles (arrow) and normal lobular architecture, confirming RTH and excluding lymphoma relapse.

Three months post-treatment, routine non-contrast chest CT revealed a *de novo*, well-circumscribed soft-tissue mass in the anterior mediastinal thymic region. Unlike the fatty thymic involution observed during chemotherapy, this mass exhibited homogeneous attenuation, a typical thymic contour, and no evidence of invasive growth (**Figure 3B**). Subsequent chest magnetic resonance imaging (MRI) indicated an anterior mediastinal mass with T2 hyperintensity and no restricted diffusion (**Figure 3C**). A re-evaluation PET-CT demonstrated mild, homogeneous ^18^F-FDG uptake confined to the thymic bed, without other hypermetabolic foci (**Figure 3D**). Although these non-invasive modalities collectively favored a benign entity, the overall clinical and imaging context in this patient created residual diagnostic ambiguity that could not be confidently resolved by non-invasive evaluation alone. Short-interval imaging follow-up and multidisciplinary conservative observation were considered; however, given these specific residual clinical and radiological uncertainties in this individual patient, CT-guided percutaneous core needle biopsy was performed to secure histological confirmation. The decision to biopsy was based on these objective findings rather than on the inability to exclude malignancy with absolute certainty. Histopathology of the biopsy specimen revealed preserved normal thymic lobular architecture with a well-differentiated cortex and medulla containing Hassall’s corpuscles (**Figure 3E**). The absence of atypical lymphoid infiltration, necrosis, or a “starry-sky” pattern confirmed RTH.

Given the aggressive biological kinetics of BL and the heightened risk of early recurrence during the initial post-treatment period, active surveillance was initiated. After multidisciplinary discussion, the following surveillance schedule was selected for this individual patient: low-dose chest CT every 3 months for the first 2 years after completion of therapy, every 6 months during years 3–5, and annually after 5 years. This schedule was chosen to balance oncological vigilance with the need to minimize cumulative radiation exposure. As of the latest follow-up—8 months following the histological confirmation of RTH—the thymic mass remained radiologically stable, demonstrating a morphological pattern entirely consistent with normal physiological development. Oncologically, the patient remains in continuous complete remission and maintains excellent overall health, with no clinical or radiological evidence of disease recurrence or treatment-related adverse events. The comprehensive diagnostic and therapeutic timeline is illustrated **Figure 4**.

**Figure 4:**
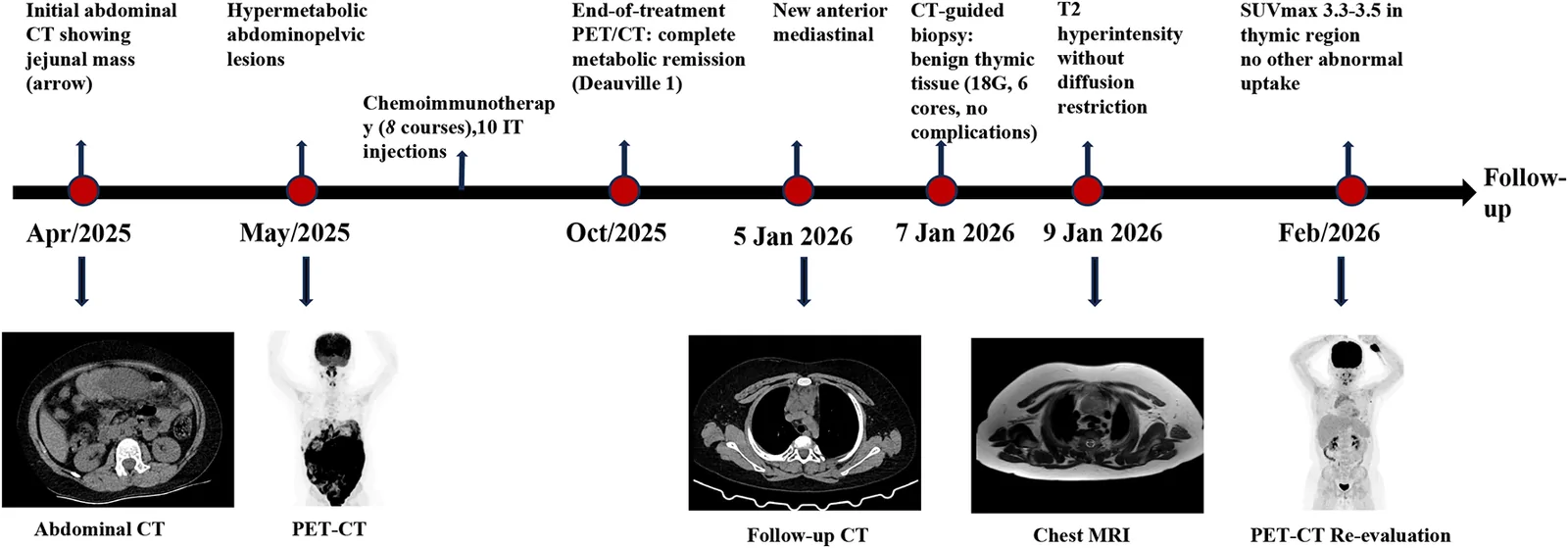
Diagnostic and therapeutic timeline illustrating the evolution from the initial presentation of jejunal Burkitt lymphoma to the detection of rebound thymic hyperplasia (RTH) mimicking mediastinal relapse.

## Discussion

The emergence of an isolated anterior mediastinal mass during routine surveillance following curative-intent therapy for primary jejunal BL presents a significant diagnostic challenge. Because BL rarely involves the mediastinum at initial presentation (1), a *de novo* mass in this region requires careful clinical evaluation to distinguish between malignant relapse and benign reactive hyperplasia (7).

Evaluating a post-treatment mediastinal mass requires a systematic differential diagnostic framework. The most common malignant etiology in children is T-lymphoblastic lymphoma (T-LBL), typically manifesting as a rapidly progressive, heterogeneous mass invading adjacent structures, often accompanied by pleural effusions and immature T-cell markers (8). Primary mediastinal large B-cell lymphoma (PMBCL) must also be excluded; however, it exhibits a distinct immune escape phenotype characterized by PD-L1 upregulation and B2M loss (9). While mediastinal involvement is a hallmark of Hodgkin lymphoma, isolated thymic relapse in HL remains exceptionally rare. In our patient, the absence of B symptoms, systemic lymphadenopathy, and abnormal peripheral blood counts strongly favored a benign etiology over disease recurrence.

Differentiating RTH from lymphoma relapse using non-invasive imaging presents notable difficulties. Morphologically, CT and MRI effectively assess lesion architecture. On CT or MRI, RTH typically appears as a single, well-defined, homogeneous mass within five months of completing treatment. Conversely, thymic relapse often manifests later in the follow-up period as a multifocal, heterogeneous, and locally invasive mass (4). Functional imaging with PET/CT introduces unique limitations. While RTH typically displays diffuse, mild-to-moderate 18F-FDG uptake, physiological thymic SUV_max_ in young patients can reach 3.73 ± 1.22—and exceed 4.0 in 44% of individuals. Relying on a rigid SUV threshold therefore carries a substantial risk of false-positive interpretations (5). Given this metabolic overlap, clinicians should approach routine post-treatment PET surveillance in pediatric NHL with caution, carefully contextualizing its utility within individual clinical scenarios, specific imaging findings, and institutional protocols to avoid misinterpretation.

When multimodal imaging cannot fully rule out malignancy, histological biopsy remains an essential diagnostic modality. However, the decision to proceed with invasive tissue sampling should remain highly individualized rather than reflexive. Clinicians must weigh the patient’s overall clinical presentation, cumulative imaging findings, and established institutional practices. As demonstrated by Qiu et al., misdiagnosing RTH as disease progression can subject pediatric patients to severe, unwarranted salvage chemotherapy (10, 11). The primary indication for tissue biopsy is persistent diagnostic ambiguity, particularly when a mass exhibits atypical imaging features, rapid growth, or emerges outside the classical temporal window for RTH within the first post-treatment year (12). In our case, a CT-guided core needle biopsy confirmed normal thymic architecture with Hassall’s corpuscles, safely averting unnecessary interventions.

To contextualize our findings, we conducted a comprehensive literature review querying PubMed, Embase, and Web of Science from database inception to June 2026, without language restrictions. Our search strategy incorporated combinations of the terms “thymic hyperplasia,” “thymic rebound,” or “thymic regrowth” with “lymphoma,” “chemotherapy,” and “Burkitt.” RTH is a recognized physiological regenerative phenomenon driven by IL-7 and Notch signaling reactivation (13), occurring in up to 67.7% of pediatric HL survivors (14). A comprehensive synthesis of previously published RTH cases and relevant pediatric lymphoma cohort studies is detailed in **Table 1** (10, 12, 15–23). As illustrated, while RTH frequently complicates post-treatment surveillance in other hematological malignancies, its manifestation following therapy for mature B-cell neoplasms like BL remains distinctly uncommon, further accentuating the unique diagnostic challenge presented by this case. Additionally, atypical presentations can confound the diagnosis. Son et al. documented ectopic thymic hyperplasia in the middle mediastinum of a 4-year-old with BL, demonstrating that regeneration is not strictly confined to the anterior compartment (15). Interestingly, thymic regeneration may serve as a radiographic surrogate for robust immune reconstitution, as patients exhibiting RTH have been associated with lower subsequent relapse rates in one exploratory study (24), a hypothesis awaiting confirmation. This inference should nonetheless be made cautiously, since peripheral lymphocyte counts and thymic output are not interchangeable measures: after lymphodepleting therapy, total CD3+ T cells may return to pre-treatment levels within one month while the reconstituted compartment remains skewed toward terminally differentiated effector-memory phenotypes with a persistently inverted CD4/CD8 ratio (25). Prospective correlation of radiographic thymic rebound with naive T-cell and recent thymic emigrant quantification—rather than absolute lymphocyte counts alone—would be required to establish RTH as a genuine surrogate for functional immune recovery.

**Table 1:** Summary of reported cases of rebound thymic hyperplasia following therapeutic interventions (2000–2025).

Reference	Age / sex	Primary diagnosis	Triggering event	Time to RTH	Diagnostic confirmation	Clinical outcome
Present Case	8 / M	Burkitt lymphoma (Stage III)	Chemoimmunotherapy (COPAD + CYM + Rituximab)	3 months	**CT/MRI:** Typical thymic contour without diffusion restriction. **PET/CT:** Mild uniform FDG uptake (SUVmax ~3.7). **Biopsy:** Normal thymic architecture.	Confirmed RTH; maintained complete remission (CR).
Son (15)	4 / M	Burkitt lymphoma	Chemotherapy (CCG-5961)	4 months	**CT:** Middle mediastinal mass. Excision: Ectopic true thymic hyperplasia.	Confirmed RTH; no evidence of lymphoma relapse.
Zhen (12), 6 Burkitt cases	2–8 / M (all male)	Burkitt lymphoma (stage III–IV)	Chemotherapy (NHL-BFM-90-B)	1–7 months	**CT:** diffuse thymic enlargement, preserved morphology. **PET/CT:** Mild uniform FDG uptake (mean SUV 2.7, range 2.3–3.5). No biopsy — imaging follow-up.	3 resolved, 3 stable at 10–16 months; all disease-free.
Tian (16), cohort	52 children	Lymphoma (incl. 17 Burkitt lymphoma)	Chemotherapy	Median 8.6 months after CR	**CT:** 81% triangular/trapezoidal, well-circumscribed, homogeneous, mild enhancement. Long-term follow-up; no biopsy.	81% spontaneous regression; 51/52 disease-free at median 32.9 months.
Aribal (17), case 1	2.5 / M	Small non-cleaved cell (B-cell) lymphoma, abdominal*	Chemotherapy (CCG-5961)	10 months	**CT:**homogeneous, well-marginated anterior mediastinal mass. Imaging follow-up.	Stable over >2 years; disease-free.
Aribal (17), case 2	6 / F	Wilms tumour	Chemotherapy (NWTS-IV) + radiotherapy	24 months	**CT:** triangular anterior mediastinal mass. Imaging follow-up.	Stable over >2 years; disease-free.
Aribal (17), case 3	16 / M	Hodgkin lymphoma, lymphocyte predominant (IIIA)	Chemotherapy (COPP alternating with ABV)	12 months	**CT:** inverted-V–shaped anterior mediastinal mass.	Stable over >2 years; disease-free.
Aribal (17), case 4	16 / F	Hodgkin lymphoma, nodular sclerosis (IIIA)	Chemotherapy (COPP/ABV)	6 months	**CT:** lobulated anterior mediastinal mass. Thoracotomy/biopsy: normal thymic lobules.	Confirmed RTH; disease-free.
Aribal (17), case 5	14 / F	Hodgkin lymphoma, mixed cellularity (IIA)	Chemotherapy (COPP/ABV)	9 months	**CT:** arc-like anterior mediastinal mass.	Stable over >2 years; disease-free.
Lin (18)	17 / M	Hodgkin lymphoma (Stage IIIsA)	Chemotherapy (ABVD)	6 months	**CT:** Recurrent mediastinal mass. Resection: Thymic hyperplasia.	Confirmed RTH; no evidence of lymphoma relapse.
Qiu (10)	YA / F	Mediastinal lymphoma	Chemotherapy + ASCT	6 months	**PET/CT:** Variable anterior mass. Biopsy: Lobular thymic tissue with fat hyperplasia.	Confirmed RTH; no disease progression.
Lázaro-García (19)	40 / F	BIA-ALCL	Chemotherapy (CHOP)	3 months	**PET/CT:** Enlarging lesion (Deauville 4). **Biopsy:** Rebound thymic hyperplasia.	Maintained CR; no signs of relapse at follow-up.
Hessissen (20)	3 / F	Nephroblastoma	Chemotherapy (VCR + Actinomycin + Adriamycin) + Surgery	4 months	**CT:** Thymus-origin mass. **FNA:** Thymic hyperplasia without malignant cells.	Spontaneous regression after 2 months; no recurrence.
Wang (21)	6 mo / M	Biliary atresia	Liver transplantation + Immunosuppression	6 months	**CT:** Homogeneous anterior mass. Thoracoscopy: True thymic hyperplasia with Hassall corpuscles.	Stable condition at 1-year follow-up.
Hwang (22)	5 mo / F	Cushing syndrome	Adrenalectomy + Hydrocortisone tapering	6 months	**CT:** Homogeneous enlarged left thymic lobe. **FNA:** Benign corticomedullary differentiation.	Spontaneous resolution shown on 1-year follow-up imaging.
Ayari (23)	49 / F	Breast cancer (Stage I)	Chemoradiotherapy + Hormonotherapy	N/A	**CT:** Triangular retrosternal mass (mixed fat/soft tissue) suggestive of thymic morphology.	Spontaneous regression; maintained CR at 7 years.

Bold indicates diagnostic confirmation methods.

The incorporation of rituximab (375 mg/m² × 4 doses) into the patient’s regimen necessitates evaluating its impact on immune reconstitution. As a CD20-targeted therapy, rituximab induces profound B-cell depletion and hypogammaglobulinemia but lacks direct toxicity against CD20-negative thymocytes and peripheral T cells (26, 27). Clinical evidence, including the Inter-B-NHL Ritux 2010 trial, confirms that rituximab addition does not significantly impair long-term T-cell or NK-cell recovery compared to standard chemotherapy (28). Consequently, the thymic rebound observed three months post-treatment most likely reflects physiological, age-dependent regeneration following glucocorticoid- and chemotherapy-induced involution, rather than a rituximab-mediated effect (29). However, because direct quantitative assessments of thymic output (e.g., sjTREC or CD31+ recent thymic emigrants) remain absent for rituximab-treated pediatric B-NHL cohorts, whether prolonged B-cell depletion indirectly modulates the magnitude of thymopoiesis warrants future prospective investigation.

As a retrospective, single-center report, this study is inherently limited by the unavailability of quantitative imaging metrics and longitudinal lymphocyte subset analyses. Consequently, a direct correlation between radiographic thymic rebound and peripheral immune reconstitution could not be established. To determine whether RTH reliably reflects functional immune recovery, future prospective studies must integrate quantitative imaging with serial immune monitoring, specifically evaluating naïve CD4+ T cells, CD31+ recent thymic emigrants, or sjTREC levels. Despite these limitations, this case underscores critical principles for post-treatment surveillance. Safely managing a *de novo* mediastinal mass in pediatric BL demands the integration of temporal onset and multimodal imaging, reserving invasive biopsies for highly equivocal cases. Accurately distinguishing benign RTH from malignant relapse is paramount to avert unwarranted therapeutic escalation and minimize treatment-related morbidity in pediatric survivors.

## Conclusion

RTH following curative-intent chemotherapy for pediatric BL is a benign regenerative phenomenon that can closely masquerade as an isolated mediastinal relapse. As supported by our 8-month serial follow-up demonstrating a radiologically stable thymic mass clinical remission, resolving this diagnostic challenge requires the careful consideration of temporal emergence patterns alongside multimodal imaging characteristics. In rare instances where non-invasive modalities yield persistently equivocal results, minimally invasive biopsy serves as a valuable diagnostic tool to confirm benign regeneration. Maintaining clinical awareness of RTH in highly ambiguous cases helps clinicians avoid unwarranted salvage interventions, supporting the long-term well-being of pediatric oncology survivors.

## Data Availability

The original contributions presented in the study are included in the article/supplementary material. Further inquiries can be directed to the corresponding authors.
